# Semi-automated analysis of HER2 immunohistochemistry in invasive breast carcinoma using whole slide images: utility for interpretation in clinical practice

**DOI:** 10.3389/pore.2024.1611826

**Published:** 2024-08-29

**Authors:** Chiu-Hsiang Connie Liao, Nilay Bakoglu, Emine Cesmecioglu, Matthew Hanna, Fresia Pareja, Hannah Y. Wen, Timothy M. D’Alfonso, Edi Brogi, Yukako Yagi, Dara S. Ross

**Affiliations:** Department of Pathology and Laboratory Medicine, Memorial Sloan Kettering Cancer Center, New York, NY, United States

**Keywords:** HER2, immunohistochemistry, breast carcinoma, whole slide imaging, semi-automated analysis

## Abstract

*Human epidermal growth factor receptor 2* (*HER2*) gene amplification and subsequent protein overexpression is a strong prognostic and predictive biomarker in invasive breast carcinoma (IBC). ASCO/CAP recommended tests for HER2 assessment include immunohistochemistry (IHC) and/or *in situ* hybridization (ISH). Accurate HER2 IHC scoring (0, 1+, 2+, 3+) is key for appropriate classification and treatment of IBC. HER2-targeted therapies, including anti-HER2 monoclonal antibodies and antibody drug conjugates (ADC), have revolutionized the treatment of HER2-positive IBC. Recently, ADC have also been approved for treatment of HER2-low (IHC 1+, IHC 2+/ISH-) advanced breast carcinoma, making a distinction between IHC 0 and 1+ crucial. In this focused study, 32 IBC with HER2 IHC scores from 0 to 3+ and HER2 FISH results formed a calibration dataset, and 77 IBC with HER2 IHC score 2+ and paired FISH results (27 amplified, 50 non-amplified) formed a validation dataset. H&E and HER2 IHC whole slide images (WSI) were scanned. Regions of interest were manually annotated and IHC scores generated by the software QuantCenter (MembraneQuant application) by 3DHISTECH Ltd. (Budapest, Hungary) and compared to the microscopic IHC score. H-scores [(3×%IHC3+) +(2×%IHC2+) +(1×%IHC1+)] were calculated for semi-automated (MembraneQuant) analysis. Concordance between microscopic IHC scoring and 3DHISTECH MembraneQuant semi-automated scoring in the calibration dataset showed a Kappa value of 0.77 (standard error 0.09). Microscopic IHC and MembraneQuant image analysis for the detection of *HER2* amplification yielded a sensitivity of 100% for both and a specificity of 56% and 61%, respectively. In the validation set of IHC 2+ cases, only 13 of 77 cases (17%) had discordant results between microscopic and MembraneQuant images, and various artifacts limiting the interpretation of HER2 IHC, including cytoplasmic/granular staining and crush artifact were noted. Semi-automated analysis using WSI and microscopic evaluation yielded similar HER2 IHC scores, demonstrating the potential utility of this tool for interpretation in clinical practice and subsequent accurate treatment. In this study, it was shown that semi-automatic HER2 IHC interpretation provides an objective approach to a test known to be quite subjective.

## Introduction

*Human epidermal growth factor receptor 2* (*HER2*) gene amplification and subsequent protein overexpression is a strong prognostic and predictive biomarker in invasive breast carcinoma (IBC), assessed by immunohistochemistry (IHC) and/or *in situ* hybridization (ISH) per the guidelines issued by the American Society of Clinical Oncology/College of American Pathologists (ASCO/CAP) [1]. HER2 IHC is generally performed first in IBC to assess for HER2 protein expression and ISH is subsequently performed in cases with equivocal IHC results [score 2+: weak to moderate complete membrane staining in >10% of tumor cells, complete membrane staining that is intense but within ≤10% of tumor cells, or moderate to intense but incomplete membrane staining (basolateral or lateral)] [1] to further assess the *HER2* gene amplification status. Although HER2 IHC is cost-effective with a fast turnaround time, this semiquantitative detection method is subjective and requires special training and expertise for accurate reporting and proper patient management [2]. The ASCO/CAP HER2 IHC scoring algorithm incorporates the percentage, completeness, intensity, and uniformity of membrane staining in IBC, with scores 0 or 1+ (negative), 2+ (equivocal) and 3+ (positive). *HER2* overexpression/amplification in IBC and the subsequent classification of HER2-positive disease is predictive of response to HER2-directed therapies in the neoadjuvant, adjuvant or metastatic settings [1]. The treatment of HER2-positive IBC has been revolutionized by the FDA approval of targeted therapies in the appropriate clinical settings, including the monoclonal antibodies trastuzumab and pertuzumab, the antibody-drug conjugates (ADC) trastuzumab emtansine (T-DM1) and fam-trastuzumab deruxtecan-nxki (T-DXd), and the tyrosine kinase inhibitors lapatinib and neratinib. Recent clinical trials have shown a benefit of ADCs in HER2 low-expressing IBC, defined as IHC 1+ or IHC 2+/ISH- [3, 4].

Targeted treatment for IBC was previously dependent upon a dichotomous HER2-positive or HER2-negative result. Now the distinction between the scores in the lower end of HER2 protein expression (IHC 0, 1+ and 2+/ISH-) becomes increasingly more crucial from a therapeutic standpoint [5]. Unfortunately, there are many challenges in HER2 testing and detection of HER2-low breast carcinoma, particularly when distinguishing between IHC scores of 0 and 1+ [6], as IHC companion diagnostic tests were optimized for the accurate detection of high HER2 protein expression levels [7]. The aim of this focused study is to validate a semi-automated computational model to assist in HER2 IHC interpretation using the QuantCenter (MembraneQuant) software developed by 3DHISTECH Ltd. (Budapest, Hungary) and provide a platform for larger studies going forward to aid in the subsequent HER2 classification and appropriate treatment of IBC.

## Materials and methods

### Case selection, HER2 testing and clinicopathologic data collection

This study was conducted under institutional review board approval (Protocol #18-013). A calibration dataset of breast biopsies and resection specimens with HER2 IHC scores 0 to 3+ and paired HER2 fluorescence *in situ* hybridization (FISH) were collected (n = 32). HER2 IHC staining (PATHWAY anti–HER2/neu [4B5], Ventana Medical Systems Inc., Tucson, Arizona, U *ultra*View PATHWAY HER2 4B5 staining procedure) was performed and clinically reported on all cases of IBC at the time of diagnosis. HER2 FISH (HER2 IQFISH pharmDx, Dako, Carpinteria, California) was performed on HER2 IHC equivocal (2+) cases in accordance with the ASCO/CAP guidelines and the standard practices at our institution. Select HER2 IHC negative (0, 1+) or positive (3+) cases were also assessed by FISH as per clinician request or for the purpose of this study. HER2 IHC and FISH were reported in accordance with the 2018 ASCO/CAP guidelines which were affirmed in the 2023 update; HER2 IHC was scored as 0, 1+, 2+, 3+ and HER2 FISH was classified into 5 groups (Group 1, HER2/CEP17 ≥2.0 and average HER2 copy number ≥4.0; Group 2, HER2/CEP17 ratio ≥2.0 and average HER2 copy number <4.0; Group 3, HER2/CEP17 ratio <2.0 and average HER2 copy number ≥6.0; Group 4, HER2/CEP17 <2.0 and average HER2 copy number ≥4.0 and <6.0; Group 5, HER2/CEP17 <2.0 and average HER2 copy number <4.0) [1]. A validation dataset of HER2 IHC 2+ cases was also collected (n = 77), consisting of IBC cases that were reported and sent for HER2 FISH between January 2021 and June 2021. The pathologic features of the cases were obtained from the clinical pathology reports.

### Semi-automated analysis

Hematoxylin and eosin (H&E) and HER2 IHC slides, with a tissue thickness of 5 microns, were scanned at 20× (0.5 um/pixel) by Leica AT2 for diagnostic use; whole slide images (WSI) were downloaded and converted into a 3DHISTECH software-compatible format. Analyses were conducted using MembraneQuant which is a function of QuantCenter by 3DHISTECH Ltd. (Budapest, Hungary). Prior to analysis, IBC regions of interest (ROI) were manually annotated by a pathologist, which included all areas of invasive carcinoma on the WSI, with areas of *in situ* carcinoma, abundant lymphoid aggregates and stromal cells avoided when possible. All annotated areas of IBC were analyzed by the software. A minimum of at least 5 to 6 tumor cells was required for analysis. The determination of the threshold for IHC scores 0, 1+, 2+, and 3+ in MembraneQuant was manually set by the study pathologist based on the calibration dataset in this study and all subsequent analyses by MembraneQuant used the same parameters and thresholds. MembraneQuant measurement parameters include membrane detection, membrane filters, nucleus detection, nucleus filters and score. Final HER2 IHC scores were generated by MembraneQuant. HER2 H-scores [(3×%IHC3+) +(2×%IHC2+) +(1×%IHC1+)] were calculated for semi-automated (MembraneQuant) analysis. Comparison of H-scores was performed using two-tailed Student’s t-test (statistical significance *p* < 0.05). On semi-automatic analysis, HER2 scores are distinguished by four colors: cells with HER2 score of 3+ are displayed in red, score of 2+ are in orange, score of 1+ are in yellow and score of 0 are in blue. The primary endpoint was the comparison of HER2 IHC microscopic/manual scoring with HER2 semi-automated scoring by MembraneQuant. Kappa values were calculated using the R studio statistical software. For the validation dataset of HER2 IHC 2+ cases, the H&E and HER2 IHC slides were scanned and analyzed using the same parameters/thresholds as in the calibration dataset. HER2 IHC scores were generated by MembraneQuant. For cases with discordant scores between MembraneQuant and microscopic scoring, the physical H&E and HER2 IHC slides were subsequently re-reviewed microscopically by three pathologists (DR, FP, TD).

## Results

**FIGURE 1:**
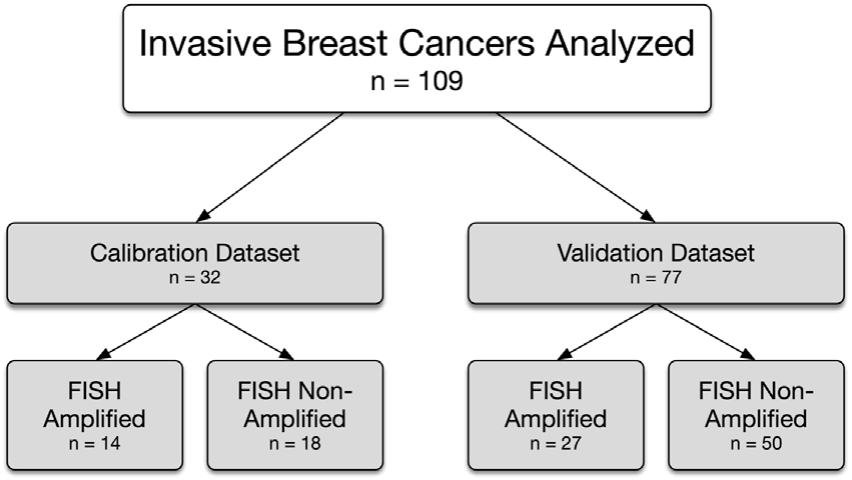
Invasive breast cancers analyzed and the HER2 FISH status.

**FIGURE 2:**
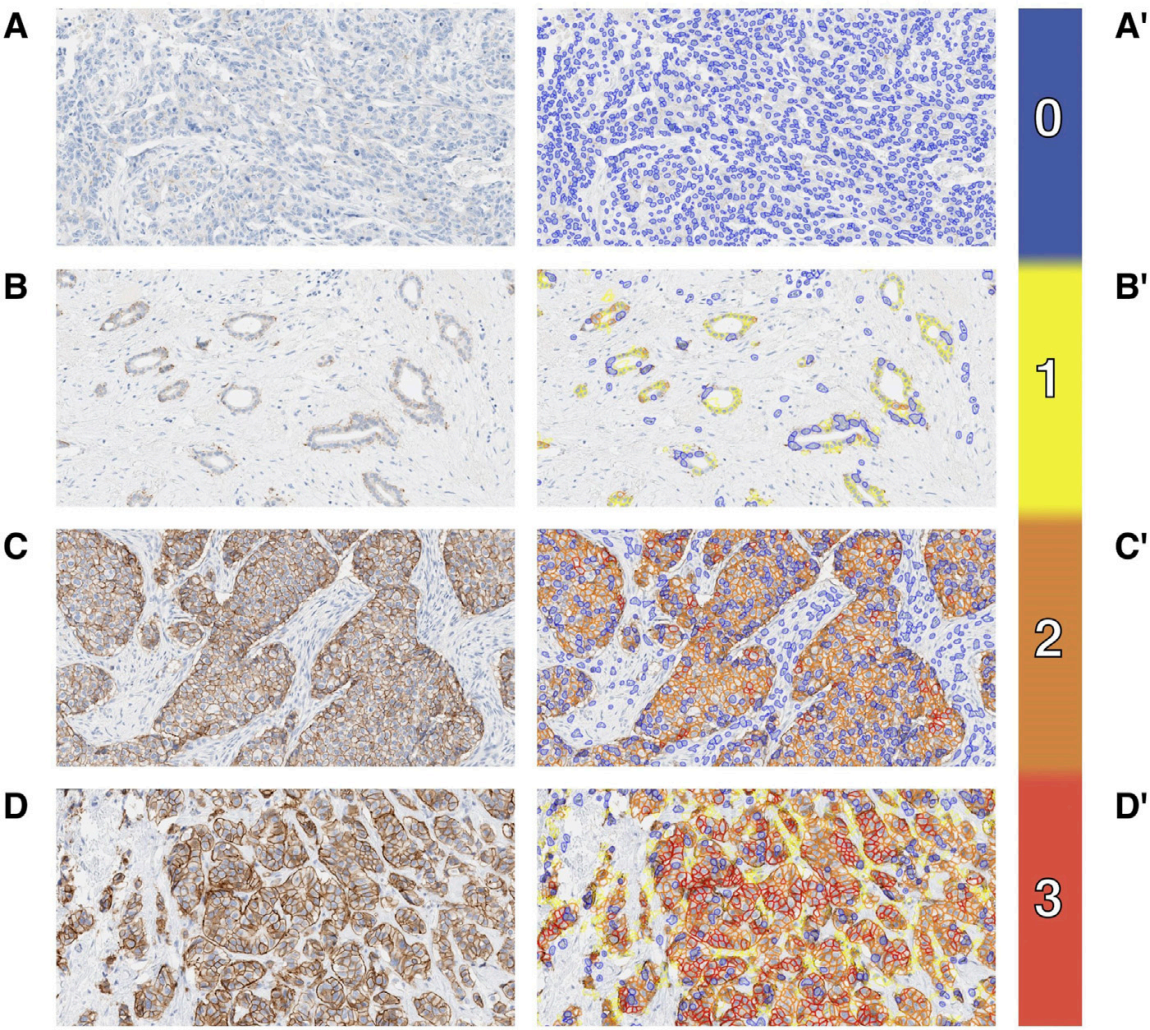
Case examples. IBC HER2 IHC with microscopic score 0 **(A)**, 1+ **(B)**, 2+ **(C)** and 3+ **(D)** and corresponding overlay from MembraneQuant by 3DHISTECH **(A′, B′, C′, D′)** with score 3 highlighted by red, score 2 orange, score 1 yellow and score 0 blue.

**FIGURE 3:**
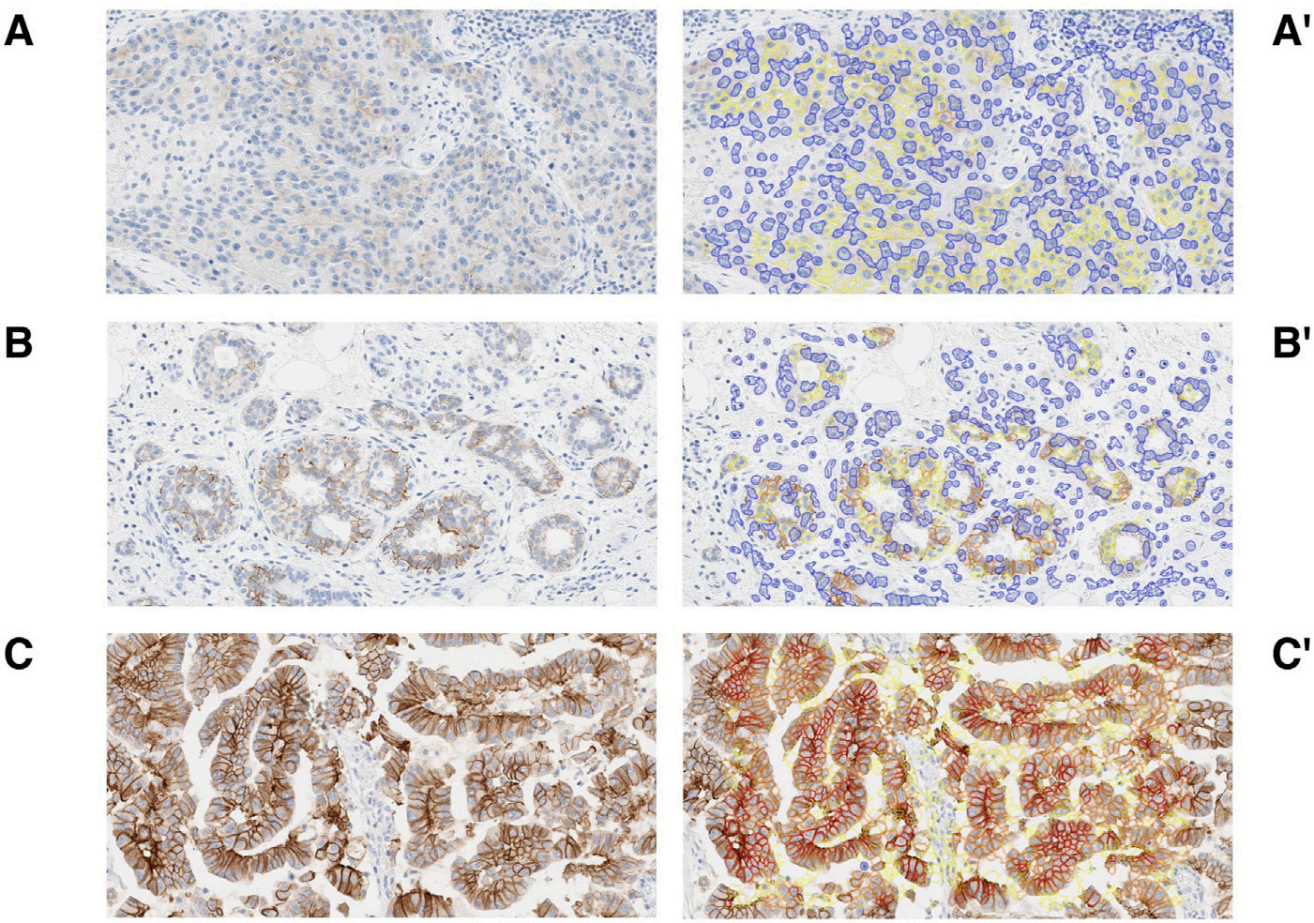
Discordant cases. Examples of discordance between microscopic IHC scoring and QuantCenter (MembraneQuant) by 3DHISTECH semi-automated scoring. Microscopic IHC 0 scored as 1+ by MembraneQuant **(A)** and corresponding overlay **(A′)**. Microscopic IHC 2+ scored as 1+ by MembraneQuant **(B)** and corresponding overlay **(B′)**. Microscopic IHC 2+ scored as 3+ by MembraneQuant **(C)** and corresponding overlay **(C′)**.

The calibration dataset was composed of 32 IBC samples (27 excisions, 5 biopsies) with both HER2 IHC and FISH results available (Figure 1). The calibration dataset included 14 HER2 amplified cases (44%) (ASCO/CAP Group 1, n = 14) and 18 HER2 non-amplified cases (56%) (ASCO/CAP Group 5, n = 17; Group 4, n = 1) by FISH. The breakdown of the HER2 IHC microscopic and MembraneQuant scores within both the HER2 amplified and non-amplified groups are shown in Table 1. The agreement between microscopic IHC and MembraneQuant scoring in the calibration dataset was found to be substantial (κ = 0.77 ± 0.09 SE). The overall concordance between the two scoring modalities is shown in Table 2 A. Of the 14 cases that were HER2 amplified by FISH, 9 cases (64%) were scored as HER2 IHC 3+ by microscopic review and were also scored as 3+ by MembraneQuant. The remaining 5 cases (36%) in the HER2 amplified group were microscopically scored as 2+; MembraneQuant scored 4 (29%) of these cases as 2+ and 1 (7%) as 3+ (Table 2 B). Out of the 18 cases that were HER2 non-amplified by FISH, 8 cases (45%) were interpreted as 2+ by microscopic scoring, while MembraneQuant scored 7 (39%) of these cases as 2+ and 1 (6%) as 1+. Four cases (22%) were scored as IHC 1+ microscopically and concordantly MembraneQuant scored all as 1+ (22%). For the 6 cases (33%) that were microscopically scored as IHC 0, 3 of these cases (16.5%) were scored as 1+ by MembraneQuant and 3 (16.5%) were scored as 0 (Table 2 C). Microscopic IHC and MembraneQuant image analysis showed a specificity of 56% and 61%, respectively, and a sensitivity of 100% for the detection of HER2 amplification (Table 3). Representative micrographs of IBCs displaying HER2 IHC scores of 0, 1+, 2+ and 3+, as well as the corresponding MembraneQuant quantification overlay are shown in Figure 2. Representative images of microscopic/MembraneQuant discordant cases (0/1+, 2+/1+ and 2+/3+) from the calibration dataset are shown in Figure 3.

**TABLE 1:** Comparison of HER2 IHC scores by microscopic and semi-automated analysis for calibration dataset (N = 32).

FISH	Microscopic HER2 IHC score				MembraneQuant HER2 IHC score			
	0	1+	2+	3+	0	1+	2+	3+
AMPLIFIED, N (%)	0 (0%)	0 (0%)	5 (16%)	9 (28%)	0 (0%)	0 (0%)	4 (12%)	10 (32%)
NON-AMPLIFIED, N (%)	6 (19%)	4 (12%)	8 (25%)	0 (0%)	3 (9%)	8 (25%)	7 (22%)	0 (0%)

**TABLE 2:** Concordance and discordance between microscopic (Mx) IHC scoring and 3DHISTECH MembraneQuant semi-automated scoring for calibration dataset (N = 32) (A). Cases are also separated based on FISH amplification status of HER2 amplified (amp) (N = 14) (B) and HER2 non-amplified (non-amp) (N = 18) (C).

A.	MembraneQuant HER2 IHC score					B.	MembraneQuant HER2 IHC score					C.	MembraneQuant HER2 IHC score				
Mx HER2 IHC Score, N = 32		0	1+	2+	3+	Mx HER2 IHC Score, N = 14 (amp)		0	1+	2+	3+	Mx HER2 IHC Score, N = 18 (non-amp)		0	1+	2+	3+
	0	3	3	0	0		0	0	0	0	0		0	3	3	0	0
	1+	0	4	0	0		1+	0	0	0	0		1+	0	4	0	0
	2+	0	1	11	1		2+	0	0	4	1		2+	0	1	7	0
	3+	0	0	0	9		3+	0	0	0	9		3+	0	0	0	0

Kappa value based on results in Table 2 A is 0.77 with standard error of 0.09.

**TABLE 3:** Confusion matrix of true positive (TP, IHC 2+ or 3+ and HER2 amplified), true negative (TN, IHC 0 or 1+, HER2 non-amplified), false positive (FP, IHC 2+ or 3+, HER2 non-amplified) and false negative (FN, IHC 0 or 1+, HER2 amplified) for microscopic (Mx) and MembraneQuant HER2 IHC score.

A.	HER2 amplification status			B.	HER2 amplification status		
Mx HER2 IHC score		Amp	Non-Amp	MembraneQuant HER2 IHC score		Amp	Non-Amp
	2+ or 3+	14	8		2+ or 3+	14	7
	0+ or 1+	0	10		0+ or 1+	0	11

The sensitivity and specificity for microscopic HER2 analysis are 100% and 56%, and for MembraneQuant are 100% and 61%.

The validation dataset was composed of 77 IBC samples, each clinically scored as HER2 IHC 2+ and subjected to HER2 FISH testing (51 excisions, 26 biopsies; Figure 1). Twenty-seven (35%) cases were HER2 amplified [ASCO/CAP Group 1, n = 24 (31%); Group 3, n = 3 (4%)] and 50 (65%) cases were HER2 non-amplified [ASCO/CAP Group 5, n = 37 (48%); Group 4, n = 12 (16%); Group 2, n = 1 (1%)] by FISH. In the HER2 amplified group (n = 27), MembraneQuant scored 19 cases (71%) as 2+ (ASCO/CAP Group 1, n = 16; Group 3, n = 3), 2 cases (7%) as 1+ (ASCO/CAP Group 1, n = 2) and 6 cases (22%) as 3+ (ASCO/CAP Group 1, n = 6); in the FISH amplified group, discordant microscopic/MembraneQuant results were observed in 8/27 (30%) cases. In the HER2 non-amplified group (n = 50), MembraneQuant scored 45 cases (90%) as 2+ (ASCO/CAP Group 5, n = 34; Group 4, n = 11), 2 cases (4%) as 1+ (ASCO/CAP Group 5, n = 2), and 3 cases (6%) as 3+ (ASCO/CAP Group 5, n = 1; Group 4, n = 1; Group 2, n = 1), with discordant microscopic/MembraneQuant results observed in 5/50 (10%) cases. Table 4 displays the HER2 IHC score called by MembraneQuant and the HER2 amplification status by FISH.

**TABLE 4:** Validation dataset (composed of HER2 IHC 2+ cases) showing the MembraneQuant IHC score by FISH amplification status (N = 77).

FISH	MembraneQuant HER2 IHC score			
	0	1+	2+	3+
AMPLIFIED, N (%)	0 (0%)	2 (2.5%)	19 (25%)	6 (8%)
NON-AMPLIFIED, N (%)	0 (0%)	2 (2.5%)	45 (58%)	3 (4%)

The semi-automated H-scores for the calibration and validation datasets are listed in Table 5. The H-score mean for the calibration dataset in the amplified group is: 135 (range 99–185) for IHC 2+ and 162 (range 88–191) for IHC 3+, whereas in the non-amplified group it is 45 (range 18–81) for IHC 1+ and 109 (range 62–157) for IHC 2+. The H-score mean for the validation dataset in the amplified group is: 128 (range 95–161) for IHC 2+, 157 (range 118–188) for IHC 3+, whereas in the non-amplified group it is 75 (56–94) for IHC 1+, 129 (82–190) for IHC 2+, and 184 (171–198) for IHC 3+. For the MembraneQuant IHC 2+ cases in the calibration dataset, no statistical significance was appreciated between the H-scores for the FISH amplified and FISH non-amplified groups (*p* = 0.27). In addition, for the FISH amplified cases in the calibration dataset, no statistical significance was appreciated between the H-scores for the MembraneQuant IHC 2+ or IHC 3+ cases (*p* = 0.18). For the FISH amplified cases in the validation dataset, there was a statistical difference between the H-scores for the MembraneQuant IHC 2+ and MembraneQuant IHC 3+ cases (*p* = 0.008). In addition, for the FISH non-amplified cases in the validation dataset, there was a statistical difference between the H-scores for the MembraneQuant IHC 2+ and MembraneQuant IHC 3+ cases (*p* = 0.00073).

**TABLE 5:** Semi-automatic H-scores generated by MembraneQuant for calibration (N = 32) and validation (N = 77) datasets.

FISH	Calibration dataset MembraneQuant HER2 IHC score				Validation dataset MembraneQuant HER2 IHC score			
	0	1+	2+	3+	0	1+	2+	3+
AMPLIFIED, N (%)	0 (0%)	0 (0%)	4 (12%)	10 (32%)	0 (0%)	2 (2.5%)	19 (25%)	6 (8%)
H-SCORE, Mean (Range)	N/A	N/A	135 (99–185)	162 (88–191)	N/A	53 (53–53)	128 (95–161)	157 (118–188)
NON-AMPLIFIED, N (%)	3 (9%)	8 (25%)	7 (22%)	0 (0%)	0 (0%)	2 (2.5%)	45 (58%)	3 (4%)
H-SCORE, Mean (Range)	0 (0–5)	45 (18–81)	109 (62–157)	N/A	N/A	75 (56–94)	129 (82–190)	184 (171–198)

**FIGURE 4:**
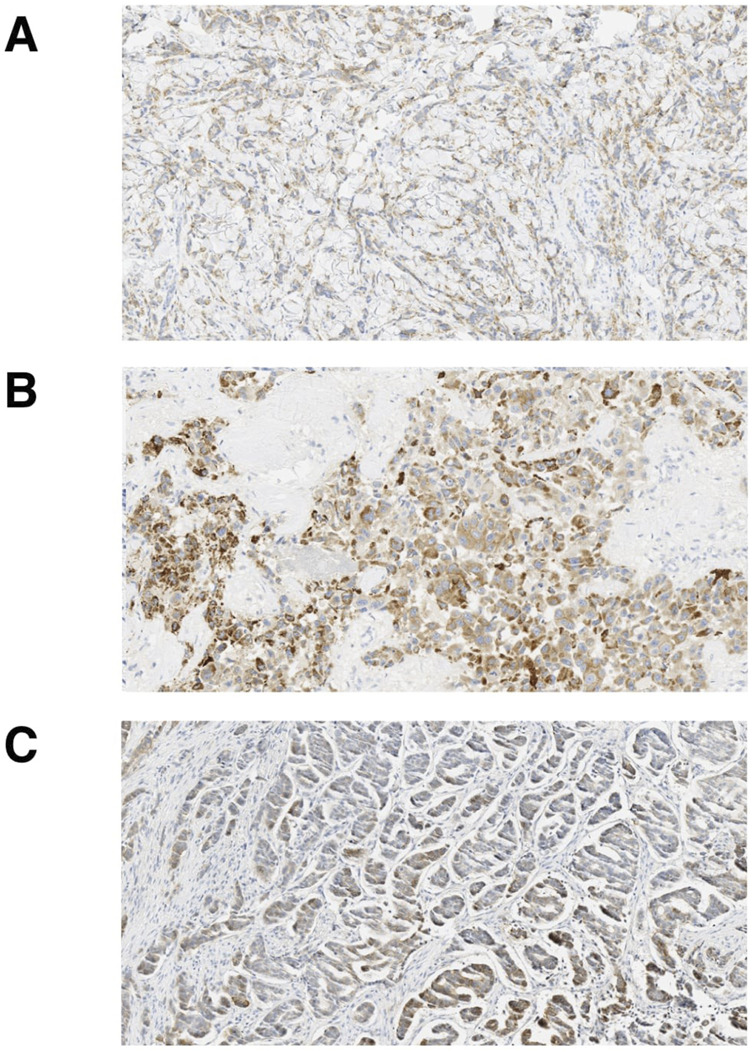
Artifacts seen with HER2 IHC staining. Examples of artifacts confounding interpretation of HER2 IHC **(A)** crush artifact, **(B)** cytoplasmic and granular staining and **(C)** chatter artifact.

The glass H&E and HER2 IHC slides of the 13 discordant microscopic/MembraneQuant cases in the validation dataset originally clinically reported as HER2 IHC 2+ were re-reviewed by three pathologists (DR, FP, TD) and their microscopic HER2 IHC scores are listed in Table 6. These 13 discordant cases included 11 primary tumors and 2 metastatic tumors. The primary tumors were all invasive breast carcinoma of no special type (ductal), including three with note of dense tumor infiltrating lymphocytes. Artifacts and limitations to analysis were appreciated during review in 8/13 discordant cases, most notably including cytoplasmic and granular IHC staining as well as areas of crush artifact. Few other limitations noted included abundant lymphocytes which impacted selection of the ROI, a case status post neoadjuvant therapy with tumor in a background of treatment effect, and a metastatic case with artifactual staining in stromal mucin associated with tumor cells. Cases with any artifacts or limitations were included in the overall analysis however notes were taken during the review. Overall, the re-review among three pathologists yielded agreement (between at least two out of three pathologists) with the original microscopic HER2 IHC score in 7 of 13 cases (54%) and MembraneQuant score in 5 of 13 cases (38%). In 11 of 13 cases (85%), MembraneQuant scored the same as at least one pathologist upon re-review. In one case, interpretation was limited by intratumoral heterogeneity (case 9, Table 6). This case was HER2 amplified by FISH and called 2+ with heterogeneity manually and 3+ by MembraneQuant. On re-review of glass slides, this case showed significant variation in the score, ranging from 0 to 2+. This case highlights the importance of ROI selection in cases where there is significant HER2 IHC staining heterogeneity. Additional images showing various artifacts limiting the interpretation of HER2 IHC, by microscopic review and MembraneQuant, including crush artifact, cytoplasmic and granular staining, and chatter from histologic sectioning, are shown in Figure 4.

**TABLE 6:** Discordant cases in the validation HER2 IHC 2+ dataset (N = 13).

Case	HER2 Score	MembQt IHC score	HER2 FISH (ASCO/CAP group)	Path 1	Path 2	Path 3	Specimen type	Histologic subtype	Limitations
1	2+	1+	AMP (Group 1)	Focal 2+	2+	1+	EX	IDC, dense TIL	Abundant TIL
2	2+	1+	AMP (Group 1)	2+	2+	1+	EX	IDC	Post NAC
3	2+	1+	NONAMP (Group 5)	1+	2+	1+	BX	IDC, dense TIL	Crush, cytoplasmic/granular staining
4	2+	1+	NONAMP (Group 5)	2+	2+	1+	BX	IDC, dense TIL	N/A
5	2+	3+	AMP (Group 1)	2+	3+	2+	EX	IDC	Crush, cytoplasmic/granular staining
6	2+	3+	AMP (Group 1)	2+	3+	3+	BX	IDC	Crush, cytoplasmic/granular staining
7	2+	3+	AMP (Group 1)	2+	3+	3+	EX	IDC	N/A
8	2+	3+	AMP (Group 1)	2+	2+	3+	BX	IDC	Crush, cytoplasmic/granular staining
9	2+	3+	AMP (Group 1)	0	2+	1-2+	EX	IDC	Intratumoral heterogeneity, crush, cytoplasmic/granular staining
10	2+	3+	AMP (Group 1)	2+	3+	3+	EX	IDC	N/A
11	2+	3+	NONAMP (Group 4)	2+	3+	3+	EX	IDC	N/A
12	2+	3+	NONAMP (Group 5)	2+	3+	2+	BX	Mucinous and micropapillary	Staining in stromal mucin
13	2+	3+	NONAMP (Group 2)	2+	2+	2+	BX	IDC	N/A

MembQt, MembraneQuant; ASCO/CAP, American Society of Clinical Oncology/College of American Pathologists; AMP, amplified; NONAMP, non-amplified; BX, biopsy; EX, excision; IDC, invasive breast carcinoma no special type (ductal); TIL, tumor infiltrating lymphocytes; NAC, neoadjuvant chemotherapy; N/A, not applicable.

## Discussion

The availability of WSI and digital pathology is becoming more widespread in clinical practice and advancements have led to the creation of ancillary software tools that can assist with interpretation and diagnosis. In this study, we sought to determine the performance of a semi-automated HER2 IHC algorithm for IHC assessment which is quite subjective in daily practice. According to Wu et al.’s systematic review and meta-analysis study on automatic evaluation of HER2 IHC status in breast cancer, the diagnostic performance of artificial intelligence (AI) shows high sensitivity and specificity with the diagnosis rendered by pathologists, and while these programs are able to distinguish between different HER2 IHC scores, the ability of AI to identify HER2 IHC scores of 2+ was slightly lower compared to recognition of scores of 0/1+ and 3+ [8]. The findings from the HER2 IHC 2+ validation dataset in our study demonstrate the wide range of HER2 IHC staining patterns that may be classified as 2+ by pathologists. The threshold for calling IHC 3+ microscopically is also variable, highlighted by the 6 HER2 amplified cases that were called 2+ on microscopic review but classified as 3+ by MembraneQuant. In clinical practice, in addition to the ASCO/CAP algorithm for evaluation, factors that impact the threshold for assigning a HER2 IHC score of 3+ include the quality of positive and negative controls, tissue quality, and staining characteristics. In the 6 HER2 amplified cases that were upgraded to 3+ by MembraneQuant, the IHC slide showed artifacts or non-membranous staining patterns in 4/6 cases, including crush and cytoplasmic/granular staining. Artifacts strongly impact a pathologist’s IHC interpretation and even with strong HER2 intensity, the threshold for calling 3+ is generally higher due to treatment implications and FISH is frequently ordered in these cases. According to ASCO/CAP guidelines, edge artifact, cytoplasmic positivity, overstaining, and misinterpretation of ductal carcinoma *in situ* (DCIS) can lead to false positive results [9]. Chatter artifacts caused when cutting on the microtome also impact interpretation. In addition, prolonged cold ischemia time, tumor heterogeneity, and improper antibody titration can lead to false negative HER2 IHC results [10]. Additionally, the performance of AI algorithms relies heavily on the quality of HER2 IHC digital images and is affected by factors such as IHC slide quality and digital slide imaging. [11, 12] These pre-analytic and analytic variables that affect the test results always need to be considered and repeat testing should be performed when indicated [1]. The study of Palm et al. further demonstrated the importance of pre-analytic variables, as it showed the agreement in HER2 IHC evaluation between AI and pathologists increased from 38.3% to 77.6% after adjustments for Ultra Cell Conditioning Solution (CC1) incubation time, antibody incubation time and counterstaining time [13]. In the one case that was classified as 1+ by MembraneQuant but was HER2 amplified by FISH in our study, the sample was status post neoadjuvant chemotherapy, with prior biopsy result of IHC 2+/FISH amplified. In the neoadjuvant setting, HER2 IHC staining has been reported to be less intense [14], either due to a true decrease in staining intensity or secondary to changes in cytomorphology. Overall, the above factors need to be taken into account when assessing IHC microscopically and especially by semi-automated analysis.

The review of the discordant HER2 IHC 2+ cohort in this study by three additional pathologists further highlights the interobserver variability in borderline cases and the need for objective tools. Distinguishing between HER2 IHC scores of 0 and 1+, which is crucial in the current HER2-low IBC era, is challenging. Fernandez et al. showed an interrater agreement lower than 70% between all HER2 IHC scores, but mostly 0 and 1+, in 19% of cases (15/80) [6]. The assessment of HER2 IHC 0 and 1+ cases was limited to the calibration dataset in this analysis with only few cases, which is a limitation of the current study. Four out of 18 non-amplified cases (22%) were scored as IHC 1+ microscopically and concordantly MembraneQuant scored all as 1+ (22%). However for the 6 out of 18 non-amplified cases (33%) that were microscopically scored as IHC 0, 3 cases were scored as 1+ by MembraneQuant and 3 were scored as 0. Distinguishing HER2 IHC 0 from 1+ remains challenging, even with computational tools, highlighting the limitations of existing HER2 assays in detecting HER2-low IBC. Wu et al. analyzed data from four studies focusing on AI’s ability to discriminate between HER2 IHC scores of 0 and 1+ and found that the performance of AI was relatively poor [8]. It is important to note that many prior concordance studies assessing digital image analysis for HER2 IHC scoring considered scores of 0 and 1+ as a single entity as, until recently, there were no clinical outcomes associated with distinguishing between these negative results [9]; Holten-Rossing et al. for example, combined 0/1+ IHC scores into a single group (HER2 negative) for analysis in their 2015 publication [15].

While MembraneQuant provides more objective parameters that are applied in a uniform manner to all slides, there are several limitations with the program. First, the software at the time of this analysis does not reliably distinguish between tumor cells and background stromal cells or tumor infiltrating lymphocytes. For this study, we used the same nuclear size cut off for each scanned slide for consistency, however adjusting the nuclear size in the program to a set threshold for individual cases may help with reducing interference from background stromal cells in the interpretation of HER2 IHC using MembraneQuant. Second, the internal scoring algorithm of MembraneQuant is weighted more heavily on the intensity of staining rather than the completeness and pattern of staining. The issue of intensity was highlighted in a separate study by Jung et al., where Al-powered ER/PR and HER2 analyzers of the breast based on a digital learning algorithm were developed, each containing both a cell detection model and a tissue segmentation model. By combining the results from both cell and tissue models, some tumor cells incorrectly detected in areas outside the ROI could be excluded. Discrepancies in the cell detection model were observed, mainly in cell classes, where the AI analyzer assessment differed by a single degree of intensity, such as changing from a HER2 IHC 2+ tumor cell to a 1+ tumor cell. In contrast, cases in which the AI analyzer misjudged the intensity by more than two grades, such as HER2 IHC 3+ tumor cell to negative (0) tumor cell, were less common [16]. Moreover, while MembraneQuant can calculate an H-score based on the fraction of cells scored with each intensity, only one overall HER2 IHC score is actually assigned. In the MembraneQuant’s calculation, scoring is purely assessed by cellularity and percentage, and the ASCO/CAP recommendation of scoring tumor cells within a contiguous area appreciable at low power [9] is not incorporated and can contribute to overscoring of HER2 IHC. In addition, a limitation of MembraneQuant is that it depends on both ROI annotation and overall H-score generation. However, most algorithms do not handle tasks with parameters beyond specific training sets due to a lack of robustness, and more importantly, they may require human intervention to annotate ROIs [17–19]. In one study by Selcuk et al., a digital learning-based method that utilizes pyramid sampling was used to automate the classification of HER2 status in IHC-stained slides. This method of analyzing features across multiple special scales addresses the challenge of HER2 expression heterogeneity without ROI selection prior to model training. The quantitative analysis involving 523 core images from 300 patients included in the study resulted in a classification accuracy of 84.70% compared to the consensus scores obtained from 5 board-certified pathologists [20].

In the present study, significant differences in H-scores were observed only in the validation set (n = 77, HER2 IHC 2+), specifically between the 2+ and 3+ scored cases by MembraneQuant, and interestingly regardless of the FISH amplification result (amplified or non-amplified), raising the question of its utility in clinical practice and alluding to some of the previously raised limitations of H-score calculation. The three MembraneQuant IHC 3+ cases that were non-amplified by FISH were notably categorized in ASCO/CAP group 2 (n = 1), group 4 (n = 1), and group 5 (n = 1). These three cases would be clinically managed as HER2-negative based on a combined FISH result and clinical IHC 2+ score, but it does highlight the need to further assess the more uncommon ASCO/CAP groups 2, 3 and 4 with semi-automated software tools. While no significant difference was observed in HER2 2+ and 3+ cases in the calibration set, this is likely due to the small number of cases evaluated. Further studies are needed to assess the value of H-scores in discriminating between cases categorized as HER2-low.

Lastly, significant time is required to run each analysis even following ROI selection. Analysis of a small biopsy at the time of this study takes on average 2–5 min and analysis of a large resection specimen takes 10–15 min. This is a significant amount of time compared to the manual review of a HER2 IHC analysis by an experienced pathologist. There is however great potential of this software for pathologists in training looking to set their own internal threshold and this should be explored further.

Previous studies have addressed issues regarding color consistency of WSI between different scanners. The inconsistency in contrast/brightness may alter visibility and impact the assessment of HER2 membrane expression as intensity/brightness is a crucial factor in scoring [21]. In regards to HER2 IHC scoring on WSI, it has been reported to show stronger intensity and higher scores than that conducted on glass slides due to possible increased color contrast on WSI [22]. However, a recent study comparing the performance of glass slides to digital slides for biomarker assessment of breast cytology specimens showed good concordance between the two methods and a good kappa correlation between glass slides and WSI for each study pathologist [23].

## Conclusion

Semi-automated analysis using WSI yielded a sensitivity of 100% and specificity of 61% in the calibration dataset and 84% of IHC 2+ cases in the validation set showed 100% agreement with microscopic analysis. In discordant cases which were also re-reviewed by three pathologists, MembraneQuant showed 85% (11/13) agreement with at least one pathologist scoring. There are inherent limitations in current HER2 tests, particularly in the detection of HER2-low IBC, that semi-automatic HER2 IHC interpretation cannot resolve at this time, however there is great value in providing an objective computational tool for a test known to be quite subjective. This software also neutralizes the experience factor in IHC scoring, raising all observers to approximately the same level and diminishing interobserver variability. Based on the results of this study, future prospective studies using semi-automated immunohistochemical analysis tools on a wider range of HER2 IHC scores are warranted.

## Data Availability

The raw data supporting the conclusions of this article will be made available by the authors, without undue reservation.
